# Application of humic acid and biofertilizers changes oil and phenolic compounds of fennel and fenugreek in intercropping systems

**DOI:** 10.1038/s41598-022-09645-4

**Published:** 2022-04-08

**Authors:** Lavin Ghaderimokri, Esmaeil Rezaei-Chiyaneh, Mahdi Ghiyasi, Mohammad Gheshlaghi, Martin Leonardo Battaglia, Kadambot H. M. Siddique

**Affiliations:** 1 Department of Plant Production and Genetics, Faculty of Agriculture, Urmia University, Urmia, Iran; 2 Department of Chromatography, Iranian Academic Center for Education Culture and Research (ACECR), Urmia, Iran; 3 Center for Sustainability Science, The Nature Conservancy, Arlington, VA USA; 4 The UWA Institute of Agriculture, The University of Western Australia, Perth, WA 6009 Australia

**Keywords:** Agroecology, Ecosystem ecology

## Abstract

The study investigated the effect of organic/biofertilizers in intercropping patterns on seed yield and yield components and essential oil, fatty acid, and phenolic compounds of fennel (*Foeniculum vulgare* L.) and fenugreek (*Trigonella foenum-graecum* L.). Experimental treatments included the application of humic acid (HA), biofertilizers (BFS), and the unfertilized control in five planting patterns [1 row fennel + 2 rows fenugreek intercropping (1F:2FG), 2 rows fennel + 2 rows fenugreek intercropping (2F:2FG), 2 rows fennel + 4 rows fenugreek intercropping (2F:4FG), and sole cropping of each species]. Sole cropping with BFS produced the highest seed yields for fennel (2233 kg ha^−1^) and fenugreek (1240 kg ha^–1^). In contrast, the 2F:2FG intercropping ratio with BFS yielded the maximum fixed oil content for fennel (17.4%) and fenugreek (8.3%). Application of HA and BFS enhanced oil yields by 66% and 75% in fennel and 40% and 57% in fenugreek, respectively. The 2F:2FG intercropping ratio with BFS produced the maximum essential oil constituents [(*E*)-anethole, estragole, and fenchone] in fennel. In addition, 2F:4FG with BFS and 1F:1FG with HA produced the highest unsaturated fatty acid (oleic and linoleic acids) concentration in both species. The 2F:2FG intercropping ratio with BFS and HA produced the highest chlorogenic acid and quercetin contents, respectively, in fennel. In contrast, the 2F:4FG intercropping ratio with HA produced the highest chlorogenic acid and caffeic acid contents in fenugreek. Intercropping fennel/fenugreek with BFS or HA improved the essential oil content (fennel only), fixed oil quality and quantity, and phenolic compounds and created a more sustainable cultivation system than sole cropping systems for both species under low-input conditions.

## Introduction

Sustainable agricultural ecosystems replace synthetic fertilizers with organic and biofertilizers^1,2^, improving soil fertility and soil health, conserving the environment, and typically increasing crop quality^3,4^. Humic acid (HA) is an environmentally friendly organic fertilizer that improves the physical, chemical, and biological parameters of soil and positively affects the quantitative and qualitative parameters of crop productivity due to its hormonal compounds^5^. Research has shown that HA stimulates seed germination in several plant species, including pepper (*Capsicum annuum*), tomato (*Solanum lycopersicum*), watermelon (*Citrullus lanatus*), lettuce (*Lactuca sativa*), borage (*Borago officinalis*), and common chicory (*Cichorium intybus*)^6,7^. In addition, the HA application enhances seed quality, increases germination percentage, rate, and uniformity, and improves seedling establishment^34^. Thus, the use of HA in intercropping systems could play a remarkable role in the clean production of medicinal plants by eliminating or significantly reducing the application of fertilizers and chemicals and their detrimental environmental outcomes^5,8^.

Biofertilizers (BFS) contain various live microorganisms, including beneficial bacteria and fungi that can convert important nutrients from unavailable to available forms, improving root expansion and seed germination^9^. An effective BFS in crop production will strengthen the plant root system and increase the yields of crops and medicinal plants^10^. Among other functions, BFS increase soil biodiversity and vital activities and help plant roots to access nutrients, especially macro elements, by increasing their solubility and enhancing environmental safety^11^.

Intercropping can increase sustainable production and reduce chemical inputs in agricultural systems^12–14^, establish an ecological balance, exploit resources to a greater extent, increase yields quantitatively and qualitatively, and decrease the damage caused by pests, diseases, and weeds^15,16^. Therefore, reducing farmer reliance on pesticides while at the same time maintaining crop productivity and quality are key intercropping goals for sustainable farming. Moreover, intercropping can increase resource use efficiency, including water and nutrients, improve soil fertility to meet plant nutrient requirements, alleviate pest and disease pressure, enhance system stability, and increase crop quantity and quality^17^. Furthermore, intercropping improves the activity of beneficial soil microbes, increasing the availability of micro- and macro-elements^8,18,19^. Studies have shown that intercropping systems are agronomically and economically viable when the intercropped species consume the resources differently. If the components of an intercropping system supplement each other and occupy their related ecological niches with minimal competition for environmental resources, such as nutrients, water, and radiation, the intercropping system is typically more productive than pure stands^15^. Legumes can play a significant role in intercropping systems because they can fix nitrogen, which is transported (directly or indirectly) from the legume component to the accompanying plants, contributing to sustainability even in a low-input system^20^. Studies have shown that intercropping with legumes and medicinal plants increases the production quantity and quality of the medicinal species versus pure stands^21–24^.

Fennel (*Foeniculum vulgare* L.) is a medicinal and aromatic plant species and a highly used member of the Apiaceae family, which comprises annual, biannual, and perennial aromatic plant species that grow up to 200 cm tall^8^. All fennel plant parts contain essential oil (EO), but the highest EO content (up to 6%) is in the fruit. The active ingredients in fennel plants are mainly used in the pharmaceutical industry to cure cough, stomachache, flatulence, and dyspepsia in children and increase milk production in mothers. In addition, fennel seeds are highly nutritious, with high protein (18–20%) and fixed oil (12–18%) contents, and a high fiber content (∼ 45%) that helps reduce glucose and cholesterol levels and regulate the levels of the cholesterol in human liver^10^.

Fenugreek (*Trigonella foenum-graecum* L.), an annual herb in the Fabaceae family, is cultivated as a medicinal, forage and vegetable crop^25^. Due to its active ingredients, fenugreek has applications in treating many diseases. In addition, its symbiotic relationship with nitrogen-fixing soil bacteria is of paramount importance in current intercropping systems aimed at reducing synthetic nitrogen fertilizer use^26^. Fenugreek seeds contain between 7 and 10% fixed oil that is rich in unsaturated fatty acids such as linoleic acid (45–47%), linolenic acid (17–21%), oleic acid (16–18%), palmitic acid (11–12%), and stearic acid (4–6%)^27^.

Most studies on BFS and organic fertilizers have focused on monoculture systems, so studies are needed to identify the effect of these fertilizers on quantitative and qualitative yields in intercropping systems. Given the significance of this issue, this study assessed the effect of BFS and HA on the quantitative and qualitative yields of fennel and fenugreek intercropped in different planting patterns. We hypothesized that: i) all intercropping patterns improve EO quality and quantity and phenolic compounds of fennel; ii) BFS and HA fertilizers in intercropping increase seed yield, essential oil and fixed oil yields, fatty acid composition, and phenolic compounds in fenugreek and fennel.

## Materials and methods

### Experimental site

The study was conducted as a factorial experiment with a randomized complete block design (RCBD) and three replications during the 2019 growing season at a farm (Long. 45° 44′ 19'' E., Lat. 36° 48′ 47'' N.) in Mahabad city in the Western Azerbaijan province, Iran. Weather data (Table 1) was collected from the Iran Meteorological Organization (https://www.irimo.ir/eng/index.php). The mean annual temperature and annual accumulated precipitation are 12 °C and 390 mm, respectively, and the elevation is 1320 m above sea level.

**Table 1:** Weather data from March to August 2019 at the study site in Mahabad, Iran.

Year	March	April	May	June	July	August
**Monthly average temperature (°C)**						
2019	9.7	14.2	22.1	25.3	26.8	22.95
10-year average	11.64	15.91	21.6	26.22	26.83	23.22
**Monthly average precipitation (mm)**						
2019	141.8	46.3	0.0	0.0	0.0	0.0
10-year average	61.1	45.7	7.9	2.0	0.6	1.9

### Treatments, land preparation, and cultivation

Prior to the study commencing, four baseline soil samples (0–30 cm depth) were taken across the experimental area to determine selected soil physicochemical properties (Table 1).

The study comprised two factors: (1) Factor 1 (‘Fertilization’, F) had three levels, including the application of (a) HA or (b) BFS and the (c) unfertilized control. The BFS treatment was a mixture comprising (i) N-fixing bacteria (NFB) containing the O4 strain of *Azotobacter vinelandii*, (ii) phosphate-solubilizing bacteria (PSB) containing the P5 strain of *Pantoea agglomerans* and the P13 strain of *Pseudomonas putida*, and (iii) K-solubilizing bacteria (KSB) containing the S14 strain of *P. koreensis* and the S19 strain of *P. vancouverensis* 5; (2) Factor 2 (‘Intercropping’, I) had five levels, including (a) 1 row fennel + 2 rows fenugreek (1F:2FG), (b) 2 rows fennel + 2 rows fenugreek (2F:2FG), (c) 2 rows fennel + 4 rows fenugreek (2F:4FG), and sole cropping of d) fennel and e) fenugreek^28^.

Fennel and fenugreek were planted at 40 cm row spacing in 4-m long rows. The on-row spacing between plants was adjusted to 10 cm for fenugreek and 25 cm for fennel, resulting in plant densities of 25 and 10 plants m^–2^, respectively^29^. Fennel and fenugreek (Urmia local landraces) seeds were obtained from the Agricultural and Natural Resources Organization of Urmia, Iran.

Seeds used in the HA fertilizer treatment were first primed in HA solution. The HA compound used in this treatment contained 62% humic acid, 8% folic acid, and 10% potassium^8^. In the BFS fertilizer treatment, fennel and fenugreek seeds were uniformly sprayed and thoroughly mixed in the shade with BF (10^8^ active bacteria per g BF) solution diluted in water^22^^.^ The HA and BFS were provided by Green Biotech Company Manufacturing, Qom, Iran.

Following the seed treatments for the HA and BFS fertilizer treatments, seeds of both plant species were air-dried at room temperature for one day and then sown on March 18, 2019. The plants were irrigated with 10 L ha^–1^ at the stem elongation and flowering stages, with the weeds removed by hand as required. No synthetic fertilizers were used in this study.

The plant material and seeds were obtained under the supervision and permission of Urmia University and according to national guidelines; all authors complied with local and national guidelines.

### Measurements

Fenugreek and fennel were harvested at the end of the growing season, on July 19, 2019 and September 5, 2019, respectively, when about 75% of the pods were yellowed. At this time, 10 plants were randomly selected from 3.2 m^2^ (2 m long × 1.6 m wide) across the four central rows in each plot to determine yield components, including plant height, pods per plant, seeds per pod, and 1000-seed weight for fenugreek and plant height, umbels per plant and 1000-seed weight for fennel.

### Fennel essential oil extraction and analysis

The EO of fennel plants was extracted by hydrodistillation in a Clevenger. For this purpose, 30 g of dried seed samples were weighed from each plot and ground to pass through a 1-mm screen. Ground samples were then placed in a jar with 300 mL water and boiled inside the Clevenger for 3 h to extract the essential oil. The extracted EO was weighed (g) and the EO content and EO yield were calculated as follows^10^:

$$\text{EO content }\left(\text{\%}\right)=\frac{\text{Extracted EO }\left(\text{\%}\right)}{30\text{ g of fennel ground seed}}\times 100$$

In addition, the EO yield of fennel (kg ha^–1^) was calculated by multiplying of EO content (%) and seed yield of fennel. Extracted EOs were dried using anhydrous sodium sulfate and stored at 4 °C until analysis.

### Essential oil analysis

The essential oils in fennel were analyzed using GC–MS (Agilent 7890/5975A GC/MSD), following the protocol of Faridvand et al. (2021)^14^.

### Fixed oil extraction

Briefly, 5 g ground seed samples of fennel and fenugreek for each treatment were mixed in 300 mL *n*-hexane to extract the fixed oil in a Soxhlet apparatus. After 6 h of extraction, the solvent was removed from the oil by rotary evaporation. The extracted oil was stored in amber glass bottles at 4 °C until the oil constituents were analyzed by GC–MS^10^. In addition, the oil yield of two plants was calculated by multiplying the oil content (%) and seed yield of fennel/fenugreek.

### Oil analysis

The fennel and fenugreek oils were analyzed using GC–MS (Agilent 7890/5975A GC/MSD), following the protocol of Rezaei-Chiyaneh et al. (2020)^10^.

### Method of extraction of phenolic acids

Dried seeds were dissolved in 2 mL of 80% MeOH and then transferred to an ultrasonic bath for 30 min. Next, the homogenates were centrifuged at 3,000 rpm for 15 min and transferred to sealed jars. Extracts were crushed through fine membrane lighters and then stored at 20 °C. Finally, 20 mL of the extract was injected into an HPLC to determine separation and analysis of phenolic acids.

### Isolation, identification, and determination of phenolic acid quantities

Analysis of phenolic acids was performed using an Agilent 1100 (HPLC) comprising 20 μL injection loop, degasser, diode-array detector (HPLC–DAD) adjusted at 250, 272, and 310 nm, four-solvent gradient pump, Octadecylsilane column, and Chemstation software for data processing. To isolate the compounds, the elution process was applied as follows: mobile phase initiated with 10% acetonitrile and 90% acetic acid (1% solution) at a flow rate of 1 mL/min, to reach 25% acetonitrile and 75% acetic acid, and 65% acetonitrile and 35% acetic acid at a flow rate of 1 mL/min after 10 min. The isolation time was 15 min.

### Land equivalent ratio (LER)

The partial LER of fennel (LER_F_) and fenugreek (LER_FG_) and total LER (LER_T_) were calculated as follows^30^:

1 $${\text{LER}}_{{\text{F}}} = \, \left( {{\text{Y}}_{{{\text{FI}}}} /{\text{ Y}}_{{{\text{FS}}}} } \right)$$

2 $${\text{LER}}_{{{\text{FG}}}} = \, \left( {{\text{Y}}_{{{\text{FGI}}}} /{\text{ Y}}_{{{\text{FGS}}}} } \right)$$

3 $${\text{LER}}_{{\text{T}}} = {\text{ LER}}_{{\text{F}}} + {\text{ LER}}_{{{\text{FG}}}}$$

where Y_FI_ and Y_FS_ represent fennel seed yield in intercropping or sole cropping, respectively, and Y_FGI_ and Y_FGS_ represent fenugreek seed yield in intercropping or sole cropping, respectively.

### Statistical analyses

Analysis of variance and mean comparisons were performed with Duncan’s multiple range test at the P < 0.05 level using the SAS 9.4 software package to assess the impact of intercropping patterns and fertilizer sources on agronomic variables and yield in fennel and fenugreek, essential oil productivity of fennel, and oil content of both species. The intercropping patterns, fertilizer sources, and their interaction were considered fixed effects, while blocks were considered random effects. All graphs were drawn in MS-Excel.

## Results

### Fennel

The main effect of fertilization (F) significantly impacted all measured parameters of fennel. Intercropping (I) pattern affected all parameters except plant height and 1000-seed weight. Significant I × F interactions occurred for umbel number, seed yield, essential oil content (EO), EO yield, oil content, and oil yield (Table 2).

**Table 2:** Analysis of variance for the effect of cropping pattern and fertilization on evaluated traits in fennel.

	Plant height	Umbel number	1000-seed weight	Seed yield	Essential oil content	Essential oil yield	Oil content	Oil yield
Block	NS	NS	NS	*	NS	NS	NS	*
Intercropping (I)	**	**	NS	**	**	**	**	**
Fertilization (F)	**	**	**	**	**	**	**	**
I × F	NS	**	NS	**	**	**	*	**

NS, *, and ** indicate non-significant differences and significant differences at the 5% and 1% probability levels, respectively.

#### Plant height

**Figure 1:**
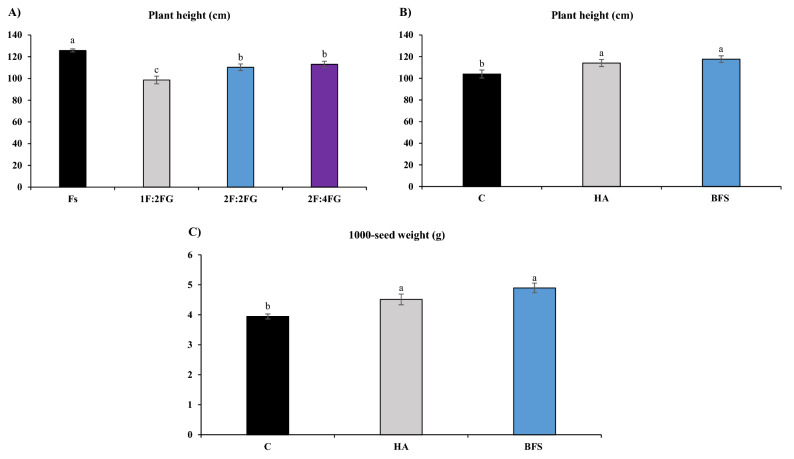
Means comparison for the main effects of cropping patterns [Fs (fennel sole cropping), 1F:2FG, 2F:2FG, 2F:4FG (ratios of fennel and fenugreek in the intercropping patterns)] on plant height (**A**), and fertilization [C (control), HA (humic acid), BFS (biofertilizers)] on plant height (**B**) and 1000-seed weight (**C**) of fennel. Different lower-case letters above the bars indicate significant (p ≤ 0.05) differences.

The tallest fennel plants (125.8 cm) occurred in the sole cropping (Fs), while the shortest plants (98.6 cm) occurred in 1F:2FG. Across intercropping patterns, the Fs treatment had 28%, 14%, 11% taller fennel plants than 1F:2FG, 2F:2FG, and 2F:4FG, respectively (Fig. 1A). Compared to the unfertilized control, HA and BFS increased fennel plant height by 10% and 13%, respectively (Fig. 1B).

#### 1000-seed weight

Compared to the unfertilized control (3.9 g per 1000 seeds), BFS and HA increased the 1000-seed weight of fennel by 24.1% and 14.5% (4.9 and 4.5 g per 1000 seeds), respectively (Fig. 1C).

#### Umbel number

**Figure 2:**
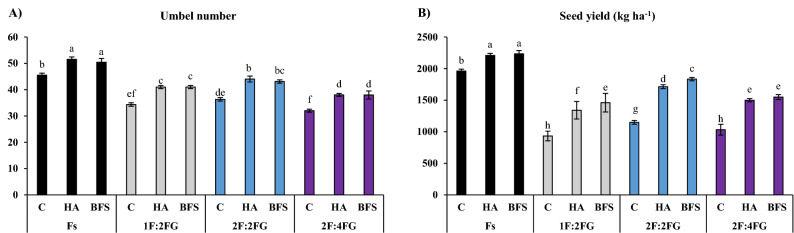
Means comparison for the interaction effect of fertilization [C (control), HA (humic acid), BFS (biofertilizers)] and different cropping patterns [Fs (fennel sole cropping), 1F:2FG, 2F:2FG, 2F:4FG (ratios of fennel and fenugreek in the intercropping patterns)] on umbel number (**A**) and seed yield (**B**) of fennel. Different lower-case letters above the bars indicate significant (p ≤ 0.05) differences.

The fennel sole cropping fertilized with HA produced the most umbels of fennel (51.5), while 2F:4FG without fertilization produced the least (32). Averaged across fertilizer types within each intercropping system, 1F:2FG, 2F:2FG, and 2F:4FG had 21.1%, 16.3%, and 26.7% fewer umbels than fennel sole cropping, respectively. Across intercropping systems, HA and BFS increased the umbel number by 17.8% and 16.5% compared with the unfertilized control, respectively (Fig. 2A).

#### Seed yield

The different intercropping patterns had lower fennel seed yields than fennel sole cropping. Sole cropping fertilized with BFS and HA produced the highest fennel seed yields (2233 and 2209 kg ha^–1^, respectively), followed by unfertilized sole cropping (1960 kg ha^–1^). The lowest seed yields occurred in the unfertilized controls in 1F:2FG (933 kg ha^–1^) and 2F:4FG (1033 kg ha^–1^). Averaged across fertilization treatments, fennel seed yield in 1F:2FG, 2F:2FG, and 2F:4FG decreased by 41.7, 26.8, and 36.3%, respectively, compared to fennel sole cropping (Fs). Averaged across intercropping patterns, HA and BFS increased fennel seed yield by 33.3% and 39.5% compared with the unfertilized control, respectively (Fig. 2B).

#### Essential oil content and yield

**Figure 3:**
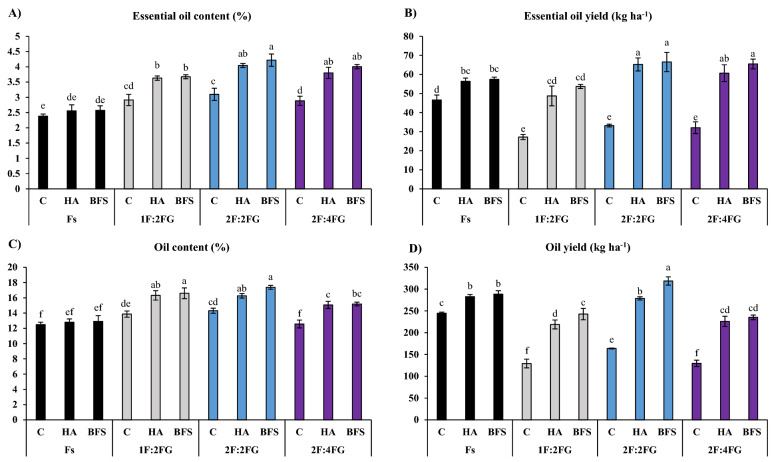
Means comparison for the interaction effect of fertilization [C (control), HA (humic acid), BFS (biofertilizers)] and different cropping patterns [Fs (fennel sole cropping), 1F:2FG, 2F:2FG, 2F:4FG (ratios of fennel and fenugreek in the intercropping patterns)] on essential oil content (**A**), essential oil yield (**B**), oil content (**C**), and oil yield (**D**) of fennel. Different lower-case letters above the bars indicate significant (p ≤ 0.05) differences.

The different intercropping patterns produced higher EO contents of fennel than fennel sole cropping. The highest absolute EO content of fennel (4.22%) occurred in 2F:2FG fertilized with BFS, although this did not statistically differ from the 2F:2FG fertilized with HA (4.04%) or 2F:4FG fertilized with HA or BFS (3.8% and 4.00%, respectively) (Fig. 3A). The lowest EO contents occurred in the unfertilized control (2.38%), HA (2.55%), and BFS (2.57%) in the Fs system. Averaged across fertilization treatments, the EO content of fennel in 1F:2FG, 2F:2FG, and 2F:4FG increased by 36%, 52%, and 44% compared to fennel sole cropping, respectively. Within each intercropping pattern, and with the exception of Fs, the HA and BFS treatments had higher EO contents of fennel, none of which significantly differed, increasing by 25% and 29%, respectively (Fig. 3A).

Maximum EO yields of fennel occurred with HA or BFS applied in 2F:2FG (65.2 and 66.6 kg ha^–1^) and 2F:4FG (60.7 and 65.5 kg ha^–1^), respectively, while the lowest EO yields occurred in the unfertilized control in 1F:2FG (27.2 kg ha^–1^), 2F:2FG (33.2 kg ha^–1^), and 2F:4FG (32.1 kg ha^–1^). Averaged across intercropping patterns, the EO yield of fennel increased by 66.1% and 74.7% with HA and BFS, respectively (Fig. 3B).

#### Fennel essential oil composition

GC–FID and GC–MS analyses identified 14 components in the fennel EO (representing 97.4–99.9% of the total composition) (Table 3), with the main constituents being trans-anethole (78.3–84.85%), estragole (3.02–7.17%), fenchone (4.14–7.52%), and limonene (3.15–4.88%). The highest percentage of (*E*)-anethole, estragole, and fenchone occurred in 2F:2FG with BFS. The highest limonene content occurred in 2F:4 FG with HA. The relative contents of trans-anethole, fenchone, and limonene increased by 3.9%, 16.6%, and 8.4% compared with fennel sole cropping. Notably, the contents of most compounds increased with HA and BFS. Compared to the unfertilized control, trans-anethole, fenchone, and limonene contents increased by 2.9%, 21.5%, and 7.9% with BFS and 2.3%, 22.4%, and 11.9% with HA, respectively (Table 3).

**Table 3:** Proportion of fennel essential oil constituents under different cropping patterns and fertilization.

Components^a^	Retention indices^b^	Treatments^c^ (%)											
		F_s_ + C	F_s_ + BFS	F_s_ + HA	1F:1FG + C	1F:1FG + BFS	1F:1FG + HA	2F + 2FG + C	2F + 2FG + BFS	2F + 2FG + HA	2F + 4FG + C	2F + 4FG + BFS	2F + 4FG + HA
Alpha-pinene	934	0.22	0.27	0.45	0.19	0.37	0.27	0.28	0.12	0.23	0.35	0.34	0.14
Camphene	949	0.02	0.01	0.03	0.02	0.05	0.01	0.02	0.03	0.01	0.03	0.06	0.01
Sabinene	973	1.1	0.13	0.17	0.08	0.15	0.11	0.12	0.07	0.11	0.14	0.12	0.11
Beta-myrcene	990	0.17	0.22	0.31	0.17	0.26	0.19	0.16	0.03	0.19	0.23	0.23	0.12
l-Phellandrene	1005	0.46	0.89	0.21	0.06	0.1	0.02	0.07	0.01	0.02	0.09	0.06	0.06
P-cymene	1022	0.08	0.04	0.09	0.06	0.01	0.07	0.02	0.08	0.06	0.05	0.04	0.01
Limonene	1030	**3.15**	**4.17**	**4.04**	**3.86**	**4.62**	**4.09**	**3.82**	**4.01**	**3.91**	**4.29**	**3.51**	**4.88**
1,8-cineole	1032	0.11	0.13	0.13	0.08	0.14	0.13	0.09	0.02	0.16	0.15	0.16	0.11
Beta-ocimene	1036	0.5	0.48	0.59	0.36	0.45	0.49	0.47	0.07	0.09	0.52	0.41	0.12
Fenchone	1091	**4.14**	**5.39**	**6.7**	**6.14**	**6.83**	**6.77**	**5.34**	**7.52**	**6.34**	**5.63**	**6.05**	**6.17**
Camphor	1148	0.14	0.14	0.16	0.14	0.16	0.15	0.1	0.01	0.18	0.15	0.16	0.12
Estragole	1200	**6.12**	**7.16**	**6.93**	**7.17**	**3.94**	**3.16**	**4.18**	**3.02**	**3.58**	**3.09**	**3.17**	**4.11**
P-anisaldehyde	1257	0.4	0.08	0.07	0.08	0.06	0.1	0.89	0.07	0.12	0.07	0.1	0.01
Trans-anethole	1293	**78.3**	**80.46**	**79.22**	**81.05**	**81.99**	**82.9**	**81.61**	**84.85**	**83.81**	**80.01**	**82.99**	**82.44**
Total identified (%)		**94.91**	**97.57**	**99.1**	**99.46**	**99.13**	**98.46**	**97.17**	**99.91**	**98.81**	**94.8**	**97.4**	**98.41**

The main components are shown in bold.

^a^Identification methods: MS, comparison of the mass spectrum with those of computer mass libraries Wiley, Adams and NIST 08; RI, comparison of retention index with those reported in Adams and NIST 08.

^b^RI, linear retention indices on DB-5 MS column, experimentally determined using homolog series of n-alkanes.

^c^C (control), BFS (biofertilizers), HA (humic acid), and Fs (fennel sole cropping), 1F:2FG, 2F:2FG, and 2F:4FG are the ratios of fennel and fenugreek in the intercropping patterns.

#### Fennel oil content and yield

Among the studied treatments, the highest fennel oil content occurred with HA or BFS application in 1F:2FG (16.3% and 16.6%) and 2F:2FG (16.3% and 17.4%), respectively. The lowest fennel oil contents occurred in the unfertilized control, HA, and BFS treatments (12.5%, 12.8%, and 12.9%, respectively) under fennel sole cropping, and the unfertilized control in 2F:4FG (12.6%). Averaged across fertilizer treatments, fennel oil content in 1F:2FG, 2F:2FG, and 2F:4FG increased by 22.8%, 26.0%, and 12.6% compared with fennel sole cropping, respectively. Across intercropping patterns, HA and BFS increased fennel oil content by 13.5% and 16.5%, respectively (Fig. 3C).

The maximum oil yield of fennel (318.6 kg ha^–1^) occurred in 2F:2FG fertilized with BFS, while the lowest oil yield (129.3 kg ha^–1^) occurred in 1F:2FG without fertilization. Across intercropping patterns, HA and BFS increased fennel oil yield by 50.8% and 62.6%, respectively (Fig. 3D).

#### Oil compounds

GC–FID and GC–MS analyses identified nine constituents that represented 94.3–97.9% of the total fennel oil composition. The main oil constituents were oleic acid (39.2–48.3%), linoleic acid (17.1–24.8%), stearic acid (10.9–15.4%), lauric acid (10.1–14.00%), and arachidic acid (2.2–3.4%). The highest oleic and linoleic acid contents occurred in 2F:4FG and 2F:2FG fertilized with BFS, respectively. Across fertilizer treatments, oleic and linoleic acid contents increased by 6% and 21%, respectively, under different intercropping patterns compared with fennel sole cropping. Across systems, HA and BFS enhanced oleic acid content by 1.8% and 8% and linoleic acid by 7.9% and 8.2%, respectively, compared with the unfertilized control. The highest percentage of stearic and lauric acids occurred in the unfertilized control of fennel sole cropping. Conversely, the lowest stearic and lauric acid contents occurred in 2F:2FG and 2F:4FG fertilized with BFS, 16.1% and 14.2% higher than fennel sole cropping, respectively. Finally, HA and BFS decreased stearic acid content by an average of 5.4% and 7.2%, respectively (Table 4).

**Table 4:** Proportion of fennel oil constituents under different cropping patterns and fertilization.

Components	Treatments^a^ (%)											
	F_s_ + C	F_s_ + BFS	F_s_ + HA	1F:1FG + C	1F:1FG + BFS	1F:1FG + HA	2F + 2FG + C	2F + 2FG + BFS	2F + 2FG + HA	2F + 4FG + C	2F + 4FG + BFS	2F + 4FG + HA
Lauric acid	**13.98**	**13.20**	**13.63**	**12.6**	**11.55**	**11.60**	**12.42**	**13.22**	**12.90**	**10.90**	**10.09**	**11.89**
Myristic acid	0.20	0.13	1.35	0.19	0.83	0.74	1.27	0.14	0.12	0.17	0.34	0.20
Palmitic acid	1.84	1.01	0.99	0.87	0.91	1.52	2.45	0.11	0.92	0.31	0.35	0.84
Stearic acid	**15.39**	**13.42**	**14.21**	**12.06**	**12.04**	**11.42**	**12.97**	**10.92**	**12.76**	**13.26**	**13.40**	**12.42**
Oleic acid	**39.24**	**42.73**	**40.76**	**40.60**	**42.89**	**41.81**	**40.66**	**44.95**	**42.76**	**45.08**	**48.29**	**43.24**
Linoleic acid	**17.08**	**19.68**	**20.84**	**22.80**	**24.38**	**23.18**	**22.78**	**24.77**	**23.69**	**21.44**	**22.19**	**23.08**
Arachidic acid	**2.69**	**3.40**	**3.27**	**2.75**	**2.33**	**3.03**	**2.58**	**2.54**	**2.41**	**2.69**	**2.25**	**2.19**
Linolenic acid	1.98	0.20	0.12	0.00	0.19	1.20	1.24	0.01	0.31	0.08	0.06	0.98
Heneicosanoic acid	1.32	1.36	0.84	0.94	0.97	1.94	1.72	0.43	1.08	0.55	0.10	1.32
Total identified (%)	**94.26**	**95.13**	**96.01**	**92.81**	**96.09**	**96.44**	**97.89**	**97.09**	**96.95**	**94.48**	**97.07**	**96.16**

The main components are shown in bold.

^a^C (control), BFS (biofertilizers), HA (humic acid), and Fs (fennel sole cropping), 1F:2FG, 2F:2FG, and 2F:4FG are the ratios of fennel and fenugreek in the intercropping patterns.

#### Phenolic compounds

The main phenolic compounds of fennel were chlorogenic acid (10.4–15.3 ppm), quercetin (7.0–17.2 ppm), and cinnamic acid (4.1–8.9 ppm). The highest chlorogenic acid and quercetin contents occurred in 2F:2FG fertilized with BFS and HA, respectively, while the lowest contents occurred in the fennel sole cropping system without fertilizer. Averaged across the three intercropping patterns, the chlorogenic acid and quercetin contents were 18.5% and 80.1% higher than the fennel sole cropping system. The chlorogenic acid and quercetin contents increased by 13% and 17% with BFS and 22% and 15% with HA, respectively (Table 5).

**Table 5:** Concentration of phenolic compounds in fennel under different cropping patterns and fertilization.

Components	Treatments^a^ (ppm)											
	F_s_ + C	F_s_ + BFS	F_s_ + HA	1F:1FG + C	1F:1FG + BFS	1F:1FG + HA	2F + 2FG + C	2F + 2FG + BFS	2F + 2FG + HA	2F + 4FG + C	2F + 4FG + BFS	2F + 4FG + HA
Gallic acid	2.19	3.27	3.05	1.41	2.46	2.67	1.65	2.93	2.72	2.01	2.88	2.90
Caffeic acid	3.90	5.04	4.00	4.021	4.55	3.98	4.68	5.98	6.11	4.90	5.78	6.44
Chlorogenic acid	10.40	11.31	11.92	12.40	12.81	13.92	12.40	15.31	14.92	10.53	12.31	14.92
Rutin	0.10	0.83	0.91	0.84	1.38	1.01	0.60	0.45	0.81	0.21	0.61	0.88
Comaric	1.27	2.60	2.170	2.499	3.298	2.922	3.397	4.061	3.417	3.909	6.126	5.070
Rosmaric acid	1.069	1.800	1.348	2.114	2.98	3.09	2.90	3.939	3.117	2.99	3.76	4.02
Quercetin	7.02	7.81	7.23	9.18	11.01	10.91	13.45	16.90	17.19	12.91	13.87	13.76
Cinamic acid	6.71	8.78	8.95	4.92	5.21	4.88	4.32	5.66	4.81	4.11	5.02	5.72
Apigenin	2.77	2.98	3.71	3.12	3.34	3.44	3.90	5.45	4.87	3.66	3.90	4.11

^a^C (control), BFS (biofertilizers), HA (humic acid), and Fs (fennel sole cropping), 1F:2FG, 2F:2FG, and 2F:4FG are the ratios of fennel and fenugreek in the intercropping patterns.

### Fenugreek

The main effects of intercropping (I) pattern (C) and fertilizer (F) were significant for all parameters analyzed in fenugreek. Significant I × F interactions occurred for plant height, pod number per plant, seed yield, oil content, and oil yield of fenugreek (Table 6).

**Table 6:** Analysis of variance for the effects of cropping patterns and fertilization on evaluated traits in fenugreek.

	Plant height	Pod number per plant	Seed number per pod	1000-seed weight	Seed yield	Oil content	Oil yield
Block	**	*	NS	*	NS	NS	NS
Intercropping (I)	**	**	**	**	**	**	**
Fertilization (F)	**	**	**	**	**	**	**
I × F	**	*	NS	NS	**	**	**

NS, *, and ** indicated non-significance differences and significant differences at the 5% and 1% probability levels, respectively.

#### Plant height

**Figure 4:**
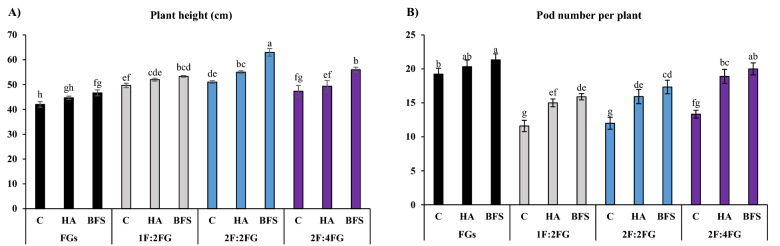
Means comparison for the interaction effect of fertilization [C (control), HA (humic acid), BFS (biofertilizers)] and different cropping patterns [FGs (fenugreek sole cropping), 1F:2FG, 2F:2FG, 2F:4FG (ratios of fennel and fenugreek in the intercropping patterns)] on plant height (**A**) and pod number per plant (**B**) of fenugreek. Different lower-case letters above the bars indicate significant (p ≤ 0.05) differences.

The 2F:2FG intercropping system fertilized with BFS produced the tallest fenugreek plants (63 cm), followed by 1F:2FG with BFS (53.3 cm) and 2F:4FG with BFS (56 cm), and 2F:2FG with HA (55 cm). The unfertilized control produced the shortest fenugreek plants (42 cm) in the sole cropping. Most fertilizer treatments across different intercropping patterns produced taller fenugreek plants than their sole cropping counterparts. Across fertilizer treatments, 1F:2FG, 2F:2FG, and 2F:4FG produced 16.2%, 26.8%, and 14.6% taller fenugreek plants than sole cropping, respectively. Across cropping patterns, BFS and HA increased fenugreek plant height by 5.7% and 15.2% compared with the unfertilized control, respectively (Fig. 4A).

#### Pod number per plant

The fenugreek sole cropping with BFS and HA and 2F:4FG with BFS produced the most pods per plant (21.3, 20.3, and 20, respectively), while the unfertilized controls in 1F:2FG, 2F:2FG, and 2F:4FG produced the least (11.6, 12, and 13.3, respectively). Across fertilization treatments, 1F:2FG, 2F:2FG, and 2F:4FG had 30.1%, 25.6%, and 14.3% fewer pods per plant, respectively, than the fenugreek sole cropping system. Across cropping systems, HA and BFS increased pod number per plant in fenugreek by 25% and 33%, respectively, relative to the corresponding sole cropping (Fig. 4B).

#### Seed number per pod

**Figure 5:**
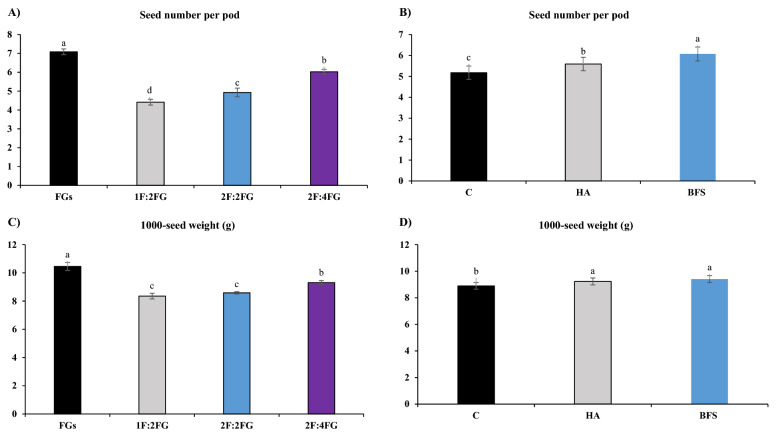
Means comparison for the main effects of cropping patterns [FGs (fenugreek sole cropping), 1F:2FG, 2F:2FG, 2F:4FG (ratios of fennel and fenugreek in the intercropping patterns)] on seed number per pod (**A**) and 1000-seed weight (**C**), and fertilization [C (control), HA (humic acid), BFS (biofertilizers)] on seed number per pod (**B**) and 1000-seed weight (**D**) of fennel. Different lower-case letters above the bars indicate significant (p ≤ 0.05) differences.

Across fertilization treatments, fenugreek sole cropping produced the most seeds per pod (7.09), followed by 2F:4FG (6.02), 2F:2FG (4.93), and 1F:2FG (4.41) (Fig. 5A). In relative terms, sole cropping produced 60.5%, 43.9%, and 17.6% more seeds per pod than 1F:2FG, 2F:2FG, and 2F:4FG (Fig. 5A). Across cropping patterns, BFS and HA increased seed number per pod by 8.1% and 17.4% compared with the unfertilized control, respectively (Fig. 5B).

#### 1000-seed weight

Among different cropping patterns, sole cropping and 1F:2FG produced the highest (10.45 g) and lowest (8.34 g) fenugreek seed weights, respectively. In relative terms, fenugreek sole cropping produced 25.3%, 21.8%, and 12.4% higher seed weights than 1F:2FG, 2F:2FG, and 2F:4FG, respectively (Fig. 5C). Across cropping patterns, BFS and HA increased fenugreek seed weight by 3.7% and 5.7% compared with the control, respectively (Fig. 5D).

#### Seed yield

**Figure 6:**
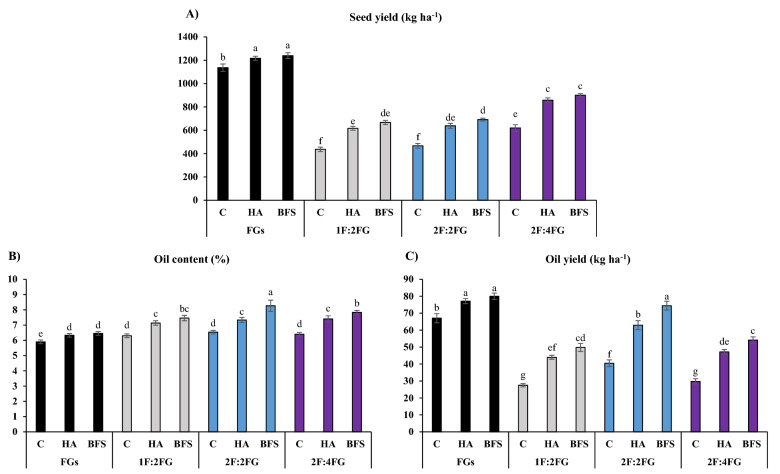
Means comparison for the interaction effects of fertilization [C (control), HA (humic acid), BFS (biofertilizers)] and different cropping patterns [FGs (fenugreek sole cropping), 1F:2FG, 2F:2FG, 2F:4FG (ratios of fennel and fenugreek in the intercropping patterns)] on seed yield (**A**), oil content (**B**), and oil yield (**C**) of fenugreek. Different lower-case letters above the bars indicate significant (p ≤ 0.05) differences.

Means comparisons showed that sole cropping produced higher fenugreek seed yields than intercropping patterns. Sole cropping with BFS (1240 kg ha^–1^) and HA (1217 kg ha^–1^) produced the highest seed yields followed by the unfertilized control (Fig. 6A). The unfertilized control in 1F:2FG (437 kg ha^–1^) and 2F:2FG (467 kg ha^–1^) produced the lowest fenugreek seed yields. In all cases, and within each cropping pattern, BFS and HS produced higher fenugreek seed yields than the unfertilized control. As a result, BFS and HA increased fenugreek seed yield by 25.2% and 31.5% compared with the unfertilized control, respectively (Fig. 6A).

#### Oil content and yield

The 2F:2FG cropping pattern with BFS produced the highest fenugreek oil content (8.3%), while the unfertilized control in sole cropping produced the lowest (5.9%). Across fertilizer treatments, 1F:2FG, 2F:2 FG, and 2F:4 FG produced 11.7%, 18.5%, and 15.7% higher fenugreek oil contents than sole cropping, respectively. In the 2F:2FG and 2F:4FG cropping patterns, BFS produced higher oil content (%) than HA. As a result, across cropping patterns, HA and BFS increased fenugreek oil content by 12.3% and 19.4%, respectively (Fig. 6B).

Sole cropping with HA and BFS and 2F:2FG with BFS produced the highest fenugreek oil yields (77.1, 80.0, and 74.4 kg ha^–1^, respectively), while the unfertilized controls in 1F:2FG and 2F:4FG produced the lowest (27.51 and 29.8 kg ha^–1^, respectively). The 1F:2FG, 2F:2FG, and 2F:4FG cropping patterns produced 45.9%, 20.7%, and 41.5% lower fenugreek oil yields than fenugreek sole cropping, respectively. Moreover, except for sole cropping, BFS produced the highest fenugreek oil yield, followed by HA and the unfertilized control (Fig. 6C).

#### Oil compounds

GC–FID and GC–MS analyses identified seven constituents (representing 91.09–99.27% of the total composition) in fenugreek oil. The main oil constituents were linoleic acid (26.1–37.1%), linolenic acid (16.9–22.4%), oleic acid (15.1–21.2%), palmitic acid (11.2–17.1%), lauric acid (5.0–12.3%), and myristic acid (3.1–6.4%). The highest linoleic and oleic acid percentages occurred in 1F:2FG and 2F:4FG with BFS. The 1F:2FG cropping pattern with BFS also had the highest linolenic acid percentage. The fenugreek sole cropping system without fertilization (control) had the lowest content of these three compounds. The intercropping patterns had 17%, 18.2%, and 17.1% higher oleic, linoleic, and linolenic acid contents than fenugreek sole cropping. In addition, HA and BFS increased oleic acid content by 15.6% and 8.8%, linoleic acid content by 12.8% and 7%, and linolenic acid content by 7.5% and 12.9%, respectively. Fenugreek sole cropping without fertilization produced the highest lauric acid and palmitic contents, 29.33% and 22.81% higher than the intercropping patterns (Table 7).

**Table 7:** Proportion of fenugreek oil constituents under different cropping patterns and fertilization.

Components	Treatments^a^ (%)											
	FG_s_ + C	FG_s_ + BFS	FG_s_ + HA	1F:1FG + C	1F:1FG + BFS	1F:1FG + HA	2F + 2FG + C	2F + 2FG + BFS	2F + 2FG + HA	2F + 4FG + C	2F + 4FG + BFS	2F + 4FG + HA
Lauric acid	**12.33**	**11.06**	**11.65**	**10.06**	**8.89**	**8.84**	**8.58**	**9.16**	**5.03**	**8.11**	**7.64**	**9.18**
Myristic acid	4.06	4.85	6.36	3.11	3.83	3.77	3.05	3.71	3.19	3.25	3.69	3.08
Palmitic acid	**17.11**	**16.05**	**16.32**	**14.09**	**12.35**	**12.96**	**12.21**	**12.94**	**11.16**	**13.09**	**12.98**	**13.96**
Stearic acid	2.94	1.17	0.86	2.96	0.9	0.82	1.98	0.89	**0.79**	1.8	0.71	0.88
Oleic acid	**15.13**	**16.88**	**16.22**	**16.96**	**17.28**	**20.55**	**16.27**	**19.41**	**19.65**	**18.62**	**19.34**	**21.21**
Linoleic acid	**26.07**	**27.24**	**26.99**	**30.52**	**32.44**	**34.06**	**29.42**	**31.66**	**37.1**	**28.15**	**30.84**	**30.55**
Linolenic acid	**16.90**	**17.11**	**17.63**	**17.02**	**22.35**	**17.91**	**19.58**	**20.53**	**22.19**	**19.18**	**22.1**	**20.41**
Total identified (%)	**93.54**	**94.36**	**96.03**	**94.72**	**98.04**	**98.91**	**91.09**	**98.30**	**99.11**	**92.20**	**97.30**	**99.27**

The main components are shown in bold.

^a^C (control), BFS (biofertilizers), HA (humic acid), and FGs (fenugreek sole cropping), 1F:2FG, 2F:2FG, and 2F:4FG are the ratios of fennel and fenugreek in the intercropping patterns.

#### Phenolic compounds

The main phenolic compounds in fenugreek were chlorogenic acid (2.01–5.49 ppm), caffeic acid (2.42–4.93 ppm), quercetin (1.98–4.45 ppm), comaric (1.09–2.43 ppm), apigenin (1.97–2.99 ppm), and gallic acid (1.76–2.92 ppm). The 2F:2FG cropping pattern with HA produced the highest quercetin and gallic acid contents, and 2F:4FG with HA produced the highest chlorogenic and caffeic acid contents. The 2F:2FG and 2F:4FG cropping patterns with BFS produced the highest comaric and apigenin contents, respectively. In contrast, fenugreek sole cropping without fertilization produced the lowest contents of the abovementioned compounds (Table 8).

**Table 8:** Proportion of fenugreek concentration of phenolic compounds under different cropping patterns and fertilization.

Components	Treatments^a^ (ppm)											
	FG_s_ + C	FG_s_ + BFS	FG_s_ + HA	1F:1FG + C	1F:1FG + BFS	1F:1FG + HA	2F + 2FG + C	2F + 2FG + BFS	2F + 2FG + HA	2F + 4FG + C	2F + 4FG + BFS	2F + 4FG + HA
Gallic acid	1.76	1.90	1.99	2.01	2.48	2.88	2.11	2.64	2.92	1.93	2.76	2.11
Caffeic acid	2.42	3.65	3.78	2.98	2.76	3.45	3.09	4.54	3.87	3.86	3.90	4.93
Chlorogenic acid	2.01	3.38	3.76	3.35	4.44	4.76	3.98	4.90	4.11	4.09	4.90	5.49
Rutin	0.17	0.90	0.54	0.21	1.01	1.81	1.10	1.23	0.87	0.76	0.90	0.93
Comaric	1.09	1.11	1.87	1.19	1.78	1.90	1.90	2.43	2.01	1.43	1.94	1.67
Rosmaric acid	0.99	1.04	1.01	1.21	1.14	1.21	1.25	1.54	1.44	1.09	1.19	1.87
Quercetin	2.90	3.09	2.99	1.98	2.93	3.01	3.12	3.87	4.45	3.66	3.87	3.94
Cinamic acid	0.89	0.65	0.90	1.09	1.11	1.18	1.01	1.19	1.70	1.45	1.43	1.56
Apigenin	1.97	2.11	2.65	1.99	2.01	2.11	1.99	2.76	2.90	2.03	2.99	2.46

^a^C (control), BFS (biofertilizers), HA (humic acid), and FGs (fenugreek sole cropping), 1F:2FG, 2F:2FG, and 2F:4FG are the ratios of fennel and fenugreek in the intercropping patterns.

### Land equivalent ratio (LER)

**Figure 7:**
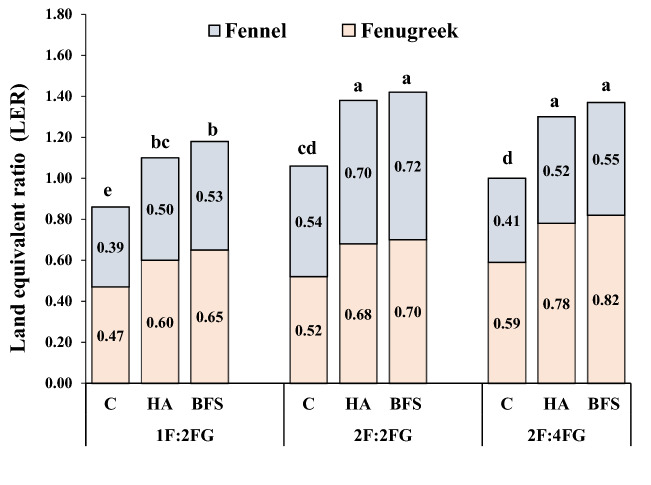
Partial and total land equivalent ratio (LER) for seed yields of different fennel and fenugreek intercropping patterns [1F:2FG, 2F:2FG, 2F:4FG (ratios of fennel and fenugreek in the intercropping patterns)] and fertilization [C (Control), HA (humic acid), BFS (biofertilizers)]. Different lower-case letters above the bars indicate significant (p ≤ 0.05) differences.

The 2F:4FG and 2F:2FG intercropping patterns treated with BFS had the highest partial LERs for fennel (0.82) and fenugreek (0.72), respectively. In addition, 2F:2FG with BFS and 1F:2FG without fertilization produced the highest (1.42) and lowest (0.86) total LERs, respectively (Fig. 7).

## Discussion

Application of humic and bio-acid fertilizers in fenugreek and fennel intercropping can improve the quantitative and qualitative yield of both species. In our field study, we calculated higher land equivalent ratios for both crops in the intercropping systems than the sole cropping systems. In our study, sole cropping produced higher growth parameter and productivity values for both plant species than intercropping. Therefore, a reduction in the values of most growth parameters and in the partial productivity of plants in intercrops could be explained by a lower number of partial plants density of each species in intercropping patterns in comparison with sole cropping systems^31^. However, comparing partial yields in sole cropping with those under intercropping does not explain the total productivity in each system. For this purpose, the LER index is a better indicator of productivity level. Parallel with our hypothesis, we calculated LER values > 1 in all intercropping patterns except for 1F:1FG without fertilization, indicating that fennel with fenugreek intercropping enhanced total system productivity compared with sole cropping. In addition, intercropping produced 6–42% higher LER values than sole cropping, implying higher productivity of both species under intercropping than sole cropping in most cases. In other words, 6–42% more land area would be needed under sole cropping to achieve the same yields under intercropping. Most intercropping patterns had partial LER values > 0.5, further highlighting the superior productivity under intercropping based on land use efficiency^32^. In sole cropping conditions, the higher intraspecific competition decreased the nutrient use efficiency of nutrients. Therefore, it can be concluded that the higher nutrient accessibility by BFS application along with improvement of environmental use efficiency and better spatial, temporal and chemical complementarity of both plants in intercropping patterns enhanced the LER index when compared with sole cropping conditions^33^. Moreover, atmospheric nitrogen fixation by the legume component of the system (fenugreek) and its direct/indirect transfer to the non-legume component (fennel) reduced the competition for inorganic nitrogen at the whole system level and enhanced plant productivity by improving nutrient absorption. Liu et al.^34^ noted that intercropping legume/non-legume species increased legume nodule formation and fixation due to the stimulation of nitrogen fixation by the non-legume and dissolution of P that acidifies the rhizosphere.

Intercropping patterns with BFS and HA generally produced higher plant productivity and LER index than unfertilized intercropping due to the positive role of BFS and HA fertilizers as nutrient suppliers for plants^20^. In line with our second hypothesis, BFS and HA improved plant growth characteristics by solubilizing and fixing nutrients for easier plant uptake, regulating hormones, and exuding plant growth regulators and phytohormones (e.g., IAA, cytokinins, GA, and ethylene)^35^. Rezaei-Chiyaneh et al.^10^ reported that BFS increased nodule number and dry weight, nitrogen fixation, and overall plant productivity in common bean. They also noted that BFS increased several plant growth parameters by reducing soil pH and improving the conditions for plant nutrient uptake. Similarly, Faridvand et al.^14^ showed that different intercropping patterns of Moldavian balm (*Dracocephalum moldavica* L.) with mung bean (*Vigna radiata* L.) had higher LER indexes than sole cropping of each species. Application of BFS and HA significantly increased the seed weight of fennel and fenugreek. Similarly, Kumari et al.^36^ reported that BFS application significantly increased the seed weight of bell pepper (*Capsicum annuum* L.).

Studies have shown that essential oil and fixed oil contents in fennel and fenugreek significantly correlate with seed yield^23^. Equally, the highest EO and fixed oil yields for both species in our study occurred in the 2F:2FG intercropping pattern treated with BFS, which had the highest EO and fixed oil contents. Consequently, any factor that increases these indices may also increase the oil yield.

In MAPs, essential oils are final terpenoid products formed by a large group of enzymes known as terpene synthases, derived from a basic structure of five carbons (C_5_H_8_), commonly called an isoprene unit, and classified depending on the number of these units in its skeleton. Biosynthesis of EO depends on the presence of different input substances and enzymes. Anethole, a monoterpene position isomer, is the main constituent of essential oils from aromatic plants, including anise, star anise, and fennel. Anethole is used as a flavoring agent in the food and pharmaceutical industries in the United States and many other countries^37^. In our study, the 2F:2FG intercropping ratio with BFS produced the highest percentage of (*E*)-anethole.

Calsamiglia et al.^38^ noted that glucose availability in plant cells, produced during photosynthesis, plays an important role in increasing terpenoid constituents in MAPs. Rostaei et al.^39^ noted that nutrient accessibility, especially N and P, in MAPs plays a key role in the development and division of cells containing EO, EO channels, glandular trichomes, and secretory ducts. Similarly, consistent with our hypothesis, Nurzynska-Wierdak et al.^40^ found that fertilizers can significantly modify the EO content and chemical constituents of MAPs, most likely associated with changes in synthesis pathways and the role of these components in plant physiology. Therefore, the higher EO content and constituents of fennel supplied with BFS in intercropping system may be due to the enhanced nutrient availability promoting enzyme activity and precursor compounds of EOs, including isoprene, phenylpropanes, and others^40,41^. Overall, EO quantity and quality of fennel improved in intercropping patterns treated with BFS. Similarly, Rezaei-Chiyaneh et al.^10^ showed that fennel/common bean intercropping with BFS improved EO content and chemical composition in terms of increased (E)-anethole, fenchone, and limonene concentrations.

In our experiment, BFS and HA increased the unsaturated fatty acid structure (FAs) and thus oil quality in most intercropping treatments. The ratio of unsaturated to saturated FAs is an important index for determining oil quality. As hypothesized, the increased concentration of unsaturated FAs in intercropping patterns treated with BFS suggests that appropriate inoculations can further enhance the health benefits of fixed oil in both fennel and fenugreek, making the seed oil more appropriate for human consumption. Likely, the higher nutrient availability in the HA and BFS treatments due to plant-growth-promoting microorganisms and atmospheric N fixation increased the photosynthetic rates of both plant species. As a result, the biosynthetic cycle of FAs may have been accelerated due to the higher supply of carbon resources (especially citrate content) and other precursor compounds, including ATP and NADPH, eventually increasing FA production^42^.

Our results showed that the phenolic compounds of fennel and fenugreek significantly increased in the 2F:2FG and 2F:4FG intercropping patterns treated with HA and BFS. In line with our second hypothesis, the biosynthesis of phenolic compounds can positively influence nutrient availability, especially N^43^. Kováčik et al.^44^ reported that phenylalanine ammonia-lyase is involved in the biosynthesis of phenolic compounds and regulated by N availability. Therefore, the higher phenolic compounds in the abovementioned intercropping treatments could be due to higher nutrient availability, with the nitrogen fixed by the legume species being transferred to the non-legume companion plants. In addition, the higher concentrations of phenolic compounds under HA and BFS were attributed to the improved nutrient availability, increasing the activity of enzymes such as phenylalanine ammonia-lyase (PAL), cinnamate 4-hydroxylase (C4H), and 4coumaryl-CoA (4CL), and phenolic contents^45^. Thus, bio/organic fertilizer application in the intercropping systems investigated in this study positively affected the formation of phenolic compounds, which could improve the overall defense mechanism of competing plants in intercropping systems.

## Conclusion

Biofertilizer and humic acid applications significantly increased fennel and fenugreek yields. of The 2F:2FG (fennel:fenugreek) intercropping ratio treated with biofertilizer was the best option in most comparisons and had the highest total land equivalent ratio index. Biofertilizer and humic acid applications increased essential oil content and essential oil yield of fennel and fixed oil content and fixed oil yield of fennel and fenugreek compared to the unfertilized control. Moreover, different intercropping patterns of fennel/fenugreek treated with biofertilizer and humic acid improved the essential oil quality of fennel and the fixed oil quality and phenolic compounds of both species. We conclude that the 2F:2FG and 2F:4FG intercropping patterns treated with biofertilizers and humic acid are excellent options for farmers looking for cleaner and more eco-friendly strategies to increase their income by improving essential oil and fixed oil quality in intercropping systems.

## Data Availability

The datasets used during the current study are available from the corresponding author on reasonable request.
